# Microbiome Changes in Connective Tissue Diseases and Vasculitis: Focus on Metabolism and Inflammation

**DOI:** 10.3390/ijms23126532

**Published:** 2022-06-10

**Authors:** Lidia La Barbera, Federica Macaluso, Serena Fasano, Giulia Grasso, Francesco Ciccia, Giuliana Guggino

**Affiliations:** 1 Department of Health Promotion, Mother and Child Care, Internal Medicine and Medical Specialties, Rheumatology Section, University of Palermo, Piazza delle Cliniche 2, 90110 Palermo, Italy; lidialb90@gmail.com (L.L.B.); giuliagr09@gmail.com (G.G.); 2 Rheumatology Unit, Department of Internal Medicine, University of Modena and Reggio Emilia, AUSL-IRCCS, Via Giovanni Amendola, 2, 42122 Reggio Emilia, Italy; federica.macaluso04@gmail.com; 3 Division of Rheumatology, Department of Precision Medicine, University of Campania Luigi Vanvitelli, S. Andrea delle Dame, Via L. De Crecchio 7, 80138 Naples, Italy; serefasa@gmail.com (S.F.); francesco.ciccia@unicampania.it (F.C.)

**Keywords:** microbiome, dysbiosis, inflammation, chronic immune-mediated inflammatory diseases

## Abstract

The microbial community acts as an active player in maintaining homeostasis and immune functions through a continuous and changeable cross-talk with the host immune system. Emerging evidence suggests that altered microbial composition, known as dysbiosis, might perturb the delicate balance between the microbiota and the immune system, triggering inflammation and potentially contributing to the pathogenesis and development of chronic inflammatory diseases. This review will summarize the current evidence about the microbiome-immunity cross-talk, especially focusing on the microbiota alterations described in patients with rheumatic diseases and on the recent findings concerning the interaction between microbiota, metabolic function, and the immune system.

## 1. Introduction

The human microbiome is defined as the whole microbes’ community, including bacteria, fungi, and protozoa and their genomes, located in the cavities and on the surfaces of the human body [1].

Over time, the microbiome and humans have co-evolved and influenced each other in a mutual process of exchange and adaptation.

The gastrointestinal (GI) tract represents the most studied and richest site, hosting many trillions of microbes, followed by cutaneous, oral, and airway sites [1].

In the healthy human GI tract, the most common enterotypes are represented by *Firmicutes, Bacteroidetes, Actinobacteria, and Proteobacteria*, with a predominant prevalence of the first two phyla, representing more than 90% of the whole gut microbial community [2,3]. In healthy conditions, the compartmentalization of this huge antigenic load into the intestinal lumen through several protective mechanisms, such as physico-chemical barriers, antimicrobial factors, and secretory IgA (sIgA) [4], minimizes its contact with the intestinal epithelial cells [5].

However, the commensal microbes are not static bystanders, and they may be considered active players, as they are crucial in maintaining homeostasis and immune functions through a continuous and changeable cross-talk with the host immune system [6,7]. Microbes produce a wide range of enzymes, chemicals, hormones, and multiple circulating metabolites that can interplay and affect the host immune system. One of the best-known mechanisms supporting this interaction is related to the chemical signaling performed by microbiota-derived metabolites [8].

**Figure 1:**
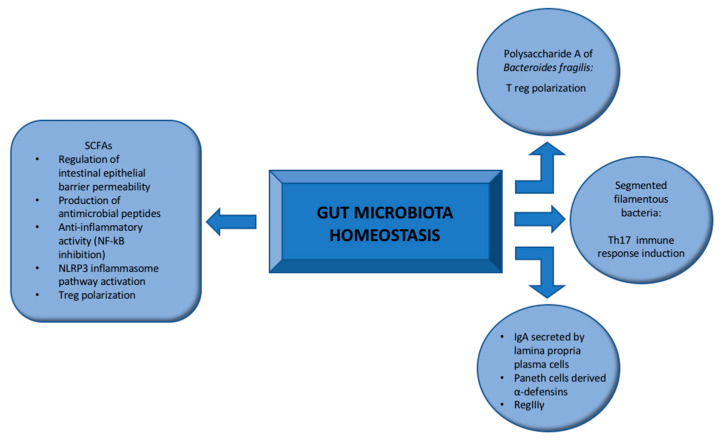
Regulation of gut microbiota homeostasis: The maintenance of intestinal microbiota homeostasis is mainly promoted by SCFAs (butyrate, propionate, and acetate), which regulate intestinal barrier permeability by orchestrating tight junction assembly and producing antimicrobial peptides. SCFAs exert immunomodulatory functions through the regulation of the innate immune response and the polarization of T cells towards a T reg phenotype. Other factors promoting intestinal homeostasis include molecules such as polysaccharide A of the commensal *Bacteroides fragilis*, IgA secreted by lamina propria plasma cells, Paneth cells derived α-defensins, and the antibacterial lectin RegIIIγ expressed in epithelial cells. Segmented filamentous bacteria tightly adhere to intestinal epithelial cells in the ileum inducing Th17-mediated immune response. NLRP3: NOD-, LRR-, and pyrin domain-containing protein 3; NF- κB: nuclear factor kappa B; SCFAs: short-chain fatty acids; Th17: T helper 17; T reg: T regulatory.

Notably, short-chain fatty acids (SCFAs), mainly butyrate, propionate, and acetate, act as Histone deacetylase (HDAC) inhibitors and G- protein-coupled receptors (GPCRs) ligands, exerting immunomodulatory functions [9] (Figure 1).

SCFAs are involved in the regulation of intestinal epithelial barrier permeability by facilitating the assembly of tight junctions and the production of antimicrobial peptides (AMP), thus contributing to the maintenance of gut homeostasis [10,11].

Several studies reported that the high intake of dietary fiber, or SCFAs produced by their fermentation, seems to be clinically helpful in the treatment and/or prevention of colitis [12,13,14,15].

Through the binding with metabolite-sensing G-protein-coupled receptor (GPR) 43 and GPR109A, SCFAs activate the NOD-, LRR-, and pyrin domain-containing protein 3 (NLRP3) inflammasome pathway, which is crucial for epithelial integrity, preventing the development of colitis in mice [16]. *Gpr43*^−/−^ mice show a severe inflammation in asthma, colitis, and arthritis models, likely related to the higher production of pro-inflammatory mediators by *Gpr43*^−/−^ immune cells and increased immune cells’ recruitment [17].

Germ-free (GF) mice that do not express SCFAs show similar immune alterations, further supporting the hypothesis that the interactions between SCFAs, especially acetate and propionate, and GPR43 markedly influence immune responses, and GPR43 may be a crucial link between diet, gut microbiota, and the inflammatory immune response [17]. 

Moreover, butyrate exerts its anti-inflammatory activity by inhibiting nuclear factor kappa B (NF-κB), decreasing leukocyte infiltration and responsiveness of intestinal macrophages to the microbiota [18,19,20] and also inducing a polarization of T cells towards a T regulatory (Treg) phenotype [18].

Other molecules such as the polysaccharide A (PSA) of the commensal *Bacteroides fragilis* may contribute to the differentiation of CD4^+^ T cells into Foxp3^+^ Treg cells [21].

Interestingly, the mono-colonization of GF mice with *Bacteroides fragilis* improved Treg anti-inflammatory functions, promoting the production of anti-inflammatory cytokines, preventing and resolving experimental colitis [22].

Other factors regulating the maintenance of the gut homeostasis include IgA secreted by lamina propria plasma cells, Paneth cells derived α-defensins, and RegIIIγ [23].

RegIIIγ is an antibacterial lectin expressed in epithelial cells that restricts bacterial-mucosal contact, avoiding immune activation [24,25,26].

Conversely, segmented filamentous bacteria (SFB) are emerging commensal microbes, tightly adhering to intestinal epithelial cells in the ileum and cells overlying Peyer’s patches, strongly related to the induction of the T helper (Th) 17 immune response [27,28].

Qualitative and/or quantitative changes in the microbiome composition, known as dysbiosis, may alter the interplay between the microbiome and the immune system, triggering inflammation and potentially contributing to the pathogenesis of chronic inflammatory disease [29,30,31,32].

Over the last decades, the link between dysbiosis and rheumatic diseases gained increasing resonance thanks to the sharp development of sequencing technologies and multiomics methodologies that have allowed a better characterization of the microbiome.

**Figure 2:**
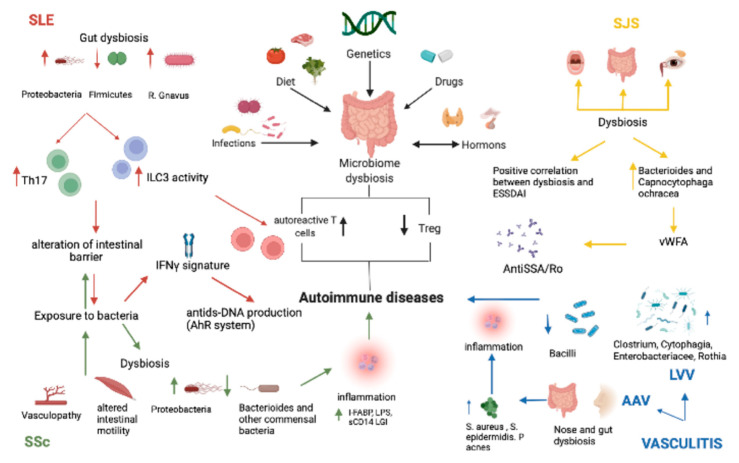
Main pathomechanisms involved in the interaction between microbiome dysbiosis and rheumatic diseases: SLE (red arrows), SJS (yellow arrows), Vasculitis (blue arrows), and SSc (green arrows). SLE: systemic lupus erythematosus; SJS: Sjogren syndrome; LVV: large vessel vasculitis; AAV: ANCA-associated vasculitis; SSc: systemic sclerosis.

This review provides an overview of the emerging literature about the involvement of dysbiosis in connective tissue diseases and vasculitis (Figure 2).

A literature search was performed with the aim of achieving a comprehensive and structured analysis of studies. The PubMed search was performed using keywords related to the diseases of interest (connective tissue diseases and vasculitis) and the main topics (microbiome, dysbiosis, and metabolome). Reference lists of all included articles and recent reviews were reviewed for their relevance to disease pathogenesis and their impact on outcome prediction and disease management.

## 2. Systemic Lupus Erythematosus

Systemic lupus erythematosus (SLE) is a systemic autoimmune disease caused by a complex interplay of genes, hormones, and environmental factors [31]. Despite recent improvements in understanding the etiopathogenesis of the disease, given its highly complex and heterogeneous nature, the pathogenesis of SLE remains unclear.

Recently, the cross-talk between commensal microbiota, metabolites, and the immune system appears to be crucial to understanding also the immunopathology of SLE. In particular, a restricted intestinal microbiota diversity and an impaired gut barrier permeability in SLE lead to immune dysregulation.

The presence of dysbiosis and the alteration of specific microorganisms, including the enrichment of Phylum Proteobacteria, are confirmed in numerous studies [33]. Interestingly, proteobacteria have been reported to be associated with intestinal inflammation [34], reflecting the inflammatory response in SLE patients. The most expanded anaerobic species of this phylum in SLE was *P. copri* [35].

Furthermore, analysis of the metabolome in serum and feces of SLE patients suggested a greater tendency to develop lipid profile disturbances [36]. In this regard, some bile acids were significantly correlated with the SLEDAI score in a multiple linear regression model, which is noteworthy considering the recent identification of the modulatory effect of these molecules on intestinal immunity [37].

The significant increase in Proteobacteria in SLE patients with respect to HC was accompanied by a decrease in Firmicutes, which did not appear to be treatment related [38].

The altered Firmicutes/Bacteroidetes ratio in SLE [39], as well as in inflammatory bowel disease [39], suggested a correlation with the shift to the immune response Th17 mediated in in vitro studies [40].

Additionally, dysbiosis could interfere with the regulatory function of ILC3 and could intensify the activity of self-reactive T cells [41].

To date, the genera significantly more represented in SLE are *Rhodococcus*, *Eggerthella*, *Klebsiella*, *Prevotella*, *Eubacterium*, *Flavonifractor*, and *Incertae sedis*; conversely, the genera *Dialister* and *Pseudobutyrivibrio* are significantly reduced [35] (Table 1).

Lupus patients have distinctive patterns of gut microbiome dysbiosis that correlate with disease activity [37]. In particular, patients with high disease activity, defined as SLEDAI ≥ 8, showed a significant reduction in microbiota diversity compared to HCs [37].

Specifically, SLE was associated with the intestinal expansion of *R. gnavus*, an obligate anaerobic species, which is directly proportional to the overall disease activity and with anti-native DNA levels, prognostic for the development of lupus nephritis [37].

In particular, a positive correlation was reported between higher levels of serum IgG anti-RG antibodies, primarily directed against antigen(s) in the *RG* strain-restricted pool of cell wall lipoglycans, the disease activity, and the occurrence of lupus nephritis, suggesting that an outgrowth of RG may affect the overall SLE pathogenesis. 

The alteration of the intestinal barrier, confirmed by the increase in total secretory IgA, fecal IgM and IgG, and calprotectin in SLE patients [37], causes immune exposure to intestinal commensal bacteria. Therefore, as a result, translocation of the bacteria to the lymph nodes and liver can drive autoimmune pathogenesis. The translocation of a commensal bacterium, *Enterococcus gallinarum*, from the small intestine to the liver in mice prone to autoimmunity, resulted in a systemic type I interferon signature and anti-dsDNA antibodies production through activation of the aryl hydrocarbon receptor (AhR) system [42].

Interestingly, the species was also positive in the liver biopsy of lupus patients [43]. These data suggest that the breakdown of the gut mucosal integrity, due to dysbiosis or genetic predispositions such as TLR deficiency, could promote the translocation of microbiota and activation of the Th17-response in SLE.

Thus, in SLE patients, the immune response to *E. gallinarum* was associated with the antibody response to a specific subset of lupus autoantigens [42]. Specifically, higher titers of anti-*E. gallinarum* were significantly associated with the presence of anti-dsDNA, anti-Sm, and anti-Ribosomal P antibodies [44]. These results provide further evidence that *E. gallinarum* may be a pathogen for SLE in susceptible individuals [42].

Some researchers hypothesized that autoimmune-susceptible individuals, who are chronically colonized by particular commensals, may develop antibodies against a bacterial ortholog that leads to autoimmunity via cross-reactivity and epitope spreading.

In line with this hypothesis, Greiling et al. demonstrated a high homology between the major T and B cell Ro60 epitopes, a well-known SLE autoantigen, and commensal bacterial Ro60 orthologs. They showed that SLE patients are colonized by orthologous Ro60-expressing commensals and sera from anti-Ro60-positive patients with SLE, but not from anti-Ro60- negative controls, and could immunoprecipitate commensal Ro60 ribonucleoproteins. Furthermore, CD4 memory T cell clones from lupus patients could be stimulated by both human Ro60 and bacterial orthologous Ro60, supporting T cell cross-reactivity [42].

## 3. Sjogren Syndrome

Sjogren syndrome (SS) is a systemic, multifactorial autoimmune disease that mainly affects exocrine glands, resulting in oral and ocular dryness, often associated with multi-organ clinical involvement [45,46].

Exocrine glands are characterized by inflammatory infiltration of dendritic cells (DCs), T and B lymphocytes that finally leads to the destruction of the acinar cells’ function. The result is the decreased secretion of salivary and lacrimal fluids, containing antimicrobial factors, the alterations of mucosal barriers, and the consequent increased risk of developing dysbiosis [47].

Several groups have been investigating the occurrence and role of dysbiosis in SS pathogenesis at different site levels.

Bacterial mimicry is one of the hypothesized mechanisms that can explain the microbiome’s involvement in disease induction [48].

To assess the hypothesis that antigens originating from oral and gut bacteria may trigger SS Antigen A (SSA)/Ro60-reactive T cells, Szymula et al. developed Ro60 reactive T cell hybridomas from HLA-DR3 transgenic mice and investigated their reactivity against oral, gut, and skin bacteria-derived antigens [49].

Ro60 reactive T cell hybridomas were activated by several peptides from commensal microbiota, also detecting the von Willebrand factor type A domain protein (vWFA) that is principally expressed by *Bacteroides intestinalis* in the gut and *Capnocytophaga ochracea* in the oral mucosa as the most potent stimulator [49].

In contrast, Lugonja et al. did not demonstrate a higher incidence of serum antibodies against ten bacteria commonly involved in periodontitis in patients with SS compared to RA and osteoarthritis (OA) patients [50].

The microbial compositions of the buccal mucosa of SS patients, non-SS sicca patients, and healthy controls were not significant different; all the groups had a higher *Firmicutes/Proteobacteria* ratio, and oral microbiome changes were related to salivary secretion and disease activity in all groups [51].

Recently, a comparison between the oral microbiome and the clinical, laboratory, and radiological features of SS patients revealed the potential role of salivary abundance of Lactobacillus and Streptococcus as biomarkers to differentiate SS patient groups from non-SS sicca patients [52].

In a study, De Paiva et al. observed intestinal dysbiosis and some mucosal alterations, such as barrier changes, a decrease in globet cells, and inflammatory infiltration, impairing after antibiotic treatment, in SS model mice more than in controls [53].

In the same study, they compared the conjunctiva, tongue, and stool microbiome of SS patients and controls, finding no significant differences in the conjunctival microbiome of the two groups, increased levels of *Lactobacillus spp*. in SS supragingival plaque samples, and *Staphylococcus aureus* and *Candida albicans* in oral mucosa and the tongue [53]. They also reported decreased diversity in the stool microbiome of SS patients (mainly defined by the reduction in *Faecalibacterium, Bacteroides, Parabacteroides,* and *Prevotella* and the increase in *Escherichia, Shigella*, and *Streptococcus* genera) and an inverse correlation between the severity of the disease and the heterogeneity of the gut microbial community [53].

The higher prevalence of severe gut dysbiosis in SS patients compared to healthy controls was further confirmed in a recent study. In the group of SS patients, the authors also found a positive correlation between the gut dysbiosis severity and higher disease activity, assessed by the scores on the European League Against Rheumatism (EULAR), Sjögren’s Syndrome Disease Activity Index (ESSDAI), and Clinical ESSDAI (ClinESSDAI), as well as increased fecal calprotectin levels and lower levels of the complement component 4 [54].

In terms of composition of the vaginal microbiome, the comparison between premenopausal SS patients experiencing vaginal dryness and healthy controls demonstrated high similarity within the two groups [4].

In addition, studies on the ocular surface (OS) microbiota in subjects with dry eyes identified characteristic differences in the abundance of some microbial species [55]. The presence of a gut-eye-tear gland microbiome axis in patients with SS is known as well as the involvement of commensal bacteria in the protection of the OS and tear glands [56]. At the phylum level, *Proteobacteria* and *Actinobacteria* were the predominant microorganisms on OS, regardless of group (SS group or dry eyes not related to SS group) [57]. Interestingly, *Acinetobacter*-related genera were abundant in the OS of SS patients, while the abundance of *Corynebacteria* was lower in the OS of SS patients [57].

Microorganisms can also induce epigenetic modifications by acting on the transcriptome in target cells and affecting their phenotype [58]. In this view, the occurrence of lymphoma as a possible complication in the course of SS may be the end result of a combination of genetics, epigenetics, dysbiosis, or latent infections through modulation of the expression of key genes [59].

Among the metabolomic studies in SS performed compared to other systemic autoimmune diseases (SAD), it was not possible to define specific metabolic footprints of SS from these works [60]. A recent study investigated the gut microbiome and plasma metabolome in patients with SAD, including SS. In line with previous observations, a depletion in tolerogenic bacteria and an increase in pathobiotic genera were observed in all SADs compared to HCs. Metabolomic analysis revealed that SADs had unique metabolomic characteristics that distinguished them from HCs. Unfortunately, it was not possible to identify a discriminating pattern for specific SAD, and the subanalysis based on Ro60/SSA positivity produced inconclusive results [61].

Primary SS is associated with an altered saliva metabolite profile, mainly defined by elevated levels of choline, taurine, and alanine, compared to HC [62]. Interestingly, low-dose doxycycline treatment normalized the levels of several metabolites in the saliva of SS patients. These metabolites mainly included dipeptides, phenylalanine, pantothenic acid, and urocanic acid and are known for their association with dysbiosis in the oral microbiota composition [63].

Therefore, the metabolomic profile in SS can potentially be used as a biological marker for monitoring response to treatment.

## 4. Systemic Sclerosis

Systemic sclerosis (SSc) is a multifactorial connective tissue disease, mostly affecting middle-aged women, characterized by vascular damage, immunological abnormalities, and fibrosis of the skin and internal organs [64].

Gastrointestinal (GI) involvement affects approximately 90% of SSc patients and can extend from the mouth to the anus [65]. Several research groups detected an altered and distinct fecal microbiome composition in SSc patients compared to HC. Whether gut dysbiosis represents a first alteration triggering the immune system or the result of the complex pathophysiology, clinical manifestations, immune dysregulation, and/or immunosuppressive treatment remains still unclear. Dysbiosis is often the consequence of altered intestinal motility, secondary to vascular disease, neuropathy, fibrosis of the intestinal wall, and atrophy of intestinal smooth muscle [66,67,68].

Intestinal dysmotility may favor small intestine bacterial overgrowth (SIBO) and systemic bacterial translocation [42,69]; vasculopathy may alter the integrity of epithelial barriers, thus inducing alterations of gut homeostasis and finally triggering a systemic immune response [70]. Gut dysbiosis in SSc patients mainly consists in the clear reduction in the commensal bacteria (*Bacteroides*, *Faecalibacterium*, and *Clostridium*) involved in the production of essential nutrients and anti-inflammatory molecules, and in the concomitant increase in pathogenic species (*Fusobacterium*, *Lactobacillus*, *g-Proteobacteria*, and also *Prevotella spp*) that promote a pro-inflammatory status [71]. Among pathogenic species, *Prevotella spp* have been mainly detected in SSc patients with severe GI involvement [71,72,73]. A decrease in the abundance of *Clostridium* was observed in patients with more severe gastrointestinal symptoms. This finding could be explained by the fact that *Clostridium* determines the expansion of Treg cells, while *Prevotella* induces an immune response mediated by Th17 cells [73]; both data can partially explain the transition to a pro-inflammatory state that occurs in patients with SSc.

The fecal microbiota and plasma metabolome of patients with SSc [74] showed the reduction in protective butyrate-producing bacteria and the increase in pro-inflammatory harmful genera. In particular, *Desulfovibrio* appears to promote intestinal damage and influence amino acid metabolism [74].

A study by Mehta et al. reported the effect of intestinal dysbiosis on the subsequent development of skin and pulmonary fibrosis in an experimental model of SSc [75]. Mice induced to have SSc-like disease received a single oral antibiotic dose (streptomycin). This drug led to a shift in the gut microbiome towards a higher *Bacteroidetes/Firmicutes* ratio, which was associated with worsening skin and lung fibrosis, compared to mice that were not given antibiotics [75].

Confirming the presence of dysbiosis, higher levels of intestinal fatty acid binding protein (I-FABP), lipopolysaccharide (LPS), and soluble CD14 (sCD14) LGI, all markers of gastrointestinal damage, were found in SSc patient sera versus HC [76].

Recent findings suggest that gut dysbiosis may be present during the early phase of the disease, before the initiation of immunosuppressive therapy, and it is associated with laboratory markers of inflammation and with both GI and extra-intestinal SSc manifestations [72,77]. Consistently, no changes were found in the absolute and relative abundances of specific genera over time within the individual subject [73].

Interestingly, dietary changes and/or intermittent antibiotic therapy may resolve SIBO, contributing to the improvement in GI symptoms and quality of life in SSc patients [78,79,80,81].

Data regarding skin microbiome alterations in SSc patients are few and controversial.

Additionally, overexpression of *Rhodotorula glutinis*, an occasional skin commensal, in lesional forearm skin biopsy samples obtained by early untreated diffuse SSc patients compared to controls was described [82].

Conversely, Johnson et al. did not observe any differences in the expression of *Rhodotorula glutinis* between SSc patients and controls. By examining lesional and non-lesional skin by RNA seq, they found substantial changes in skin microbial composition between SSc patients and HC, mainly characterized by a decrease in lipophilic taxa (*Propionibacterium* and *Staphylococcus*) and a concomitant enrichment in Gram-negative taxa, including *Burkholderia*, *Citrobacter*, and *Vibrio*. These differences did not correlate with disease duration and/or disease subtype. Moreover, lesional and non-lesional skin showed a similar microbial composition [83]. A comparison between the microbial communities of samples from limited cutaneous (lc)SSc and diffuse cutaneous (dc)SSc patients showed a lower richness in those affected by dcSSc. In particular, a significant depletion of *Coprococcus* was observed in dcSSc compared with lcSSc [84].

The lung microbiome and its correlation to SSc-Interstitial Lung Disease (ILD) have not been investigated yet. It would be interesting to explore the relationship between dysbiosis and ILD onset and/or progression.

Future research is needed to assess the role of the microbiome in the SSc pathogenesis and the underlying mechanisms involved.

## 5. Large Vessels Vasculitis

Giant Cell Arteritis (GCA) and Takayasu Arteritis (TAK) are the two main large vessel vasculitides (LVV), affecting the aorta and its major branches [85].

Emerging data suggesting the presence of differences in the microbial community in LVV patients are recently increasing. To date, no single pathogen characteristic of GCA or TAK has been identified.

Recently, Hoffman et al. investigated the temporal artery microbiome in GCA using fluorescent in situ hybridization (FISH) and 16S rRNA sequencing. The largest differential abundances seen between GCA and non-GCA temporal arteries include *Proteobacteria, Bifidobacterium, Parasutterella*, and *Granulicatella.* No microbiomal differences were found between biopsy-positive and biopsy-negative GCA [86].

This study showed an over-representation of the phylum *Firmicutes* and an under-representation of *Proteobacteria* and *Actinobacteria* in the temporal arteries of patients with GCA compared to controls [86]. Notably, the microbiome of biopsy-negative GCA patients was similar to that of biopsy-positive GCA patients, suggesting a potential underlying similarity in temporal arteries of GCA patients not reflected in histopathologic changes [86].

A decreased microbiome diversity was also reported in inflammatory thoracic aortic aneurysms of patients with aortitis, including patients with GCA and clinically isolated aortitis (CIA), compared to HC [87]. Specifically, an over-representation of the genera *Phascolarctobacterium* and *Rothia* and the family *Enterobacteriaceae* and a reduction in genera protective against inflammation on mucosal surfaces, such as *Prevotella*, *Acinetobacter*, *Klebsiella*, *Staphylococcus*, and *Corynebacterium*, in the aortitis group compared to HC have been described [87].

Interestingly, Desbois et al. recently demonstrated specific alterations in the blood microbiome in LVV patients compared with healthy controls, also describing differences between TAK and GCA patients [88]. Notably, TAK patients showed higher levels of *Clostridia, Cytophagia*, and *Deltaproteobacteria* and a decrease in *Bacilli* compared with controls. Active TAK patients had significantly lower levels of Staphylococcus than inactive TAK patients [88]. Microbiota of TAK compared with GCA patients were found to show higher levels of *Candidatus Aquiluna* and *Cloacibacterium*. In patients with TAK, differences in the blood microbiome were also associated with specific metabolic functions [88]. These alterations were associated with enrichment of specific metabolic pathways, which may be involved in LVV pathogenesis. However, further studies are necessary to assess these alterations in a larger group of patients and to correlate microbiome changes with immune system alterations.

## 6. ANCA-Associated Vasculitis (AAV)

Emerging data also reported microbiome changes, at different sites, in ANCA-associated vasculitis (AAV). Analysis of the nasal and upper respiratory microbiome in patients with granulomatosis with polyangiitis (GPA) highlighted the role of *Staphylococcus aureus* in the pathogenesis of the disease. The nasal carriage of *S. aureus* has an increased risk of disease recurrence, which may explain the advantage of antibiotic treatment with trimethoprim/sulfamethoxazole in preventing relapse in localized disease [89,90]. Ree et al. confirmed S aureus among the most abundant species identified in nasal swabs of GPA, followed by the lower relative abundance of *P. acnes* and *Staphylococcus epidermidis.* Interestingly, after immunosuppressive therapy, there was a reduction in the abundance of *P acnes*, *P granulosum*, and *S. epidermidis* observed mainly in patients with GPA not on immunosuppressive therapy [91].

A comparison of the nasal microbiota in GPA and RA showed a reduced abundance of bacterial taxa and microbial richness in the GPA samples. Significantly more colonization of S. aureus was observed in the nasal microbiome of GPA compared to RA and healthy control samples [92].

Furthermore, Najem et al. [93] described gut dysbiosis in patients affected by active AAV compared with remission AAV patients. Particularly, they found an enrichment of *Dialister* and *Prevotella* taxa in active AAV patients and a positive correlation between worsening gut dysbiosis and the Birmingham Vasculitis Activity Score for Wegener Granulomatosis (BVAS/WG).

## 7. Potential Strategies of Gut Microbiota Modulation

Recently, the modulation of dysbiosis for therapeutic purposes is gaining an increasing resonance. Interestingly, a correlation between Mediterranean diet and increased levels of SCFA has been observed, highlighting a potential dietary intervention on microbiome homeostasis [94].

Another well-established mechanism of gut microbiota modulation consists in using probiotics (living bacteria) or prebiotics (substances promoting the growth of specific bacteria). Recently, there has also been increasing attention on the fecal microbiota transplant (FMT), a technique capable of reversing dysbiosis, leading to a potential improvement in disease activity.

FMT is effective in treating *Clostridium difficile* colitis, and there are some encouraging results from its application in inflammatory bowel disease (IBD) [95,96,97].

An emerging interest is also addressed to pharmacomicrobiomics, or the drug-microbiome interaction. The intestinal microbial complex may modulate both the pharmacokinetics and the pharmacodynamics of drugs, directly and/or indirectly. This phenomenon could partially explain the remarkable interindividual variability in the therapeutic response, especially in the rheumatology field [98].

The aforementioned mechanisms may be considered promising tools to modulate the microbiome, potentially representing a new therapeutic approach along with pharmacologic intervention to limit inflammation and achieve better control of rheumatic diseases.

Regarding standard treatments for the diseases under investigation, only a few studies have conducted sub-analyses that stratify patients based on therapeutic interventions. Although there is evidence in the literature of specific changes in the intestinal microbiota in renal transplant patients treated with mycophenolate mofetil [99] and the ability of certain intestinal bacteria to metabolize azathioprine [100], suggesting a pharmacomicrobial approach to optimize IBD therapy, their possible role in rheumatologic diseases has to be fully elucidated.

Methotrexate may also influence the community of intestinal microorganisms, regulating their diversity and function. It inhibits the growth of intestinal bacterial strains in a dose-dependent manner, which results in reduced stimulation of immune system activation and decreased tolerance to Treg cell induction [101]. In addition, the use of hydroxychloroquine in SLE appears to reduce the abundance of *Enterobacteriaceae* [102]. Concerning other standard treatment options, no significant data have emerged to date.

## 8. Conclusions

Over the last decades, the study of the human microbiome and its functional potential in health and diseases gained increasing resonance.

The accumulating data highlight an intricate interplay and equilibrium between the microbiome and the harboring host and the consequent influence of microbiome alterations and related metabolic pathways on the host immune system.

Although there are some conflicting results, most of the emerging literature shows the presence of alterations in the microbial community composition at different sites in patients with rheumatic diseases compared to controls, thus supporting the hypothesis that dysbiosis in concert with metabolic imbalance can lead to inflammation. Therefore, the strength of our work is to analyze the interaction between the microbiota and the immune system, providing insights to discover new therapeutic targets and potential biomarkers of disease. However, the main limitation concerns the lack of adequate stratification of patients into subgroups based on clinical and laboratory characteristics and therapeutic interventions. Furthermore, it is not yet established whether microbiota changes are primary or secondary to local and systemic immune disturbances.

Currently, the main challenge is identifying a “specific microbiomic and metabolomic profile” for each rheumatic disease and its underlying etiopathogenetic mechanism, revealing factors that may influence the disease onset and/or progression and opening new therapeutic scenarios.

## Figures and Tables

**Table 1:** The table reports the main studies investigating on the gut microbiome composition in systemic lupus erythematosus.

		References
**Increased microbial communities**	*Prevotella (P. copri)*, *Rhodococcus*, *Eggerthella*, *Klebsiella*, *Eubacterium*, *Flavonifractor*, *Incertae sedis*, *Ruminococcus (R. gnavus)*	[35,37,39]
**Decreased** **microbial communities**	*Dialister*, *Pseudobutyrivibrio*	[35]
